# Automated detection of the head-twitch response using wavelet scalograms and a deep convolutional neural network

**DOI:** 10.1038/s41598-020-65264-x

**Published:** 2020-05-20

**Authors:** Adam L. Halberstadt

**Affiliations:** 1 Department of Psychiatry, University of California San Diego, La Jolla, CA USA; 2 Research Service, VA San Diego Healthcare System, San Diego, CA USA

**Keywords:** Biological techniques, Computational biology and bioinformatics, Neuroscience

## Abstract

Hallucinogens induce the head-twitch response (HTR), a rapid reciprocal head movement, in mice. Although head twitches are usually identified by direct observation, they can also be assessed using a head-mounted magnet and a magnetometer. Procedures have been developed to automate the analysis of magnetometer recordings by detecting events that match the frequency, duration, and amplitude of the HTR. However, there is considerable variability in the features of head twitches, and behaviors such as jumping have similar characteristics, reducing the reliability of these methods. We have developed an automated method that can detect head twitches unambiguously, without relying on features in the amplitude-time domain. To detect the behavior, events are transformed into a visual representation in the time-frequency domain (a scalogram), deep features are extracted using the pretrained convolutional neural network (CNN) ResNet-50, and then the images are classified using a Support Vector Machine (SVM) algorithm. These procedures were used to analyze recordings from 237 mice containing 11,312 HTR. After transformation to scalograms, the multistage CNN-SVM approach detected 11,244 (99.4%) of the HTR. The procedures were insensitive to other behaviors, including jumping and seizures. Deep learning based on scalograms can be used to automate HTR detection with robust sensitivity and reliability.

## Introduction

Serotonergic hallucinogens such as psilocybin, *d*-lysergic acid diethylamide (LSD), and mescaline can induce profound alterations of consciousness, including changes in thought, perception, and mood. The psychedelic effects produced by these drugs are thought to be primarily mediated by activation of the 5-HT_2A_ receptor^1–4^. In recent years, studies have explored the potential therapeutic effects of serotonergic hallucinogens in disorders such as anxiety and depression^5–8^. Given the potential therapeutic efficacy of these compounds, there is an increasing need for preclinical behavioral models that can be used to study the pharmacology and mechanism of action of hallucinogens. Administration of LSD and other hallucinogens to mice induces the head-twitch response (HTR), a paroxysmal side-to-side head rotation, which is mediated via 5‐HT_2A_ receptor activation^9,10^. The HTR assay is a popular behavioral model for assessing 5-HT_2A_ activation by hallucinogens in rodents^11–15^. Although the non-hallucinogenic LSD analog lisuride is active in some preclinical behavioral models used to study hallucinogens, lisuride does not induce the HTR in mice^16,17^. In addition, there is a robust correlation between the potency of hallucinogens in the HTR paradigm and their activity in humans^18^.

The HTR has traditionally been assessed by direct observation, making data collection time-consuming and subjective. To increase the reliability and throughput of HTR studies, the behavior can be detected using a head-mounted magnet and a magnetometer coil^17^. The head movement made during the HTR is highly rhythmic and has a higher frequency than most other spontaneous behaviors. Each HTR is recorded as a sinusoidal wavelet with a characteristic frequency (80–100 Hz, sometimes with a 40–50 Hz subharmonic) and duration (<0.15 s)^17,19^. With suitable filtering, head twitches can be discriminated from other behaviors with a high degree of sensitivity and reliability. These methods have been used to study the *in vivo* response to a number of novel hallucinogens and other 5-HT_2A_ receptor agonists^19–26^.

**Figure 1:**
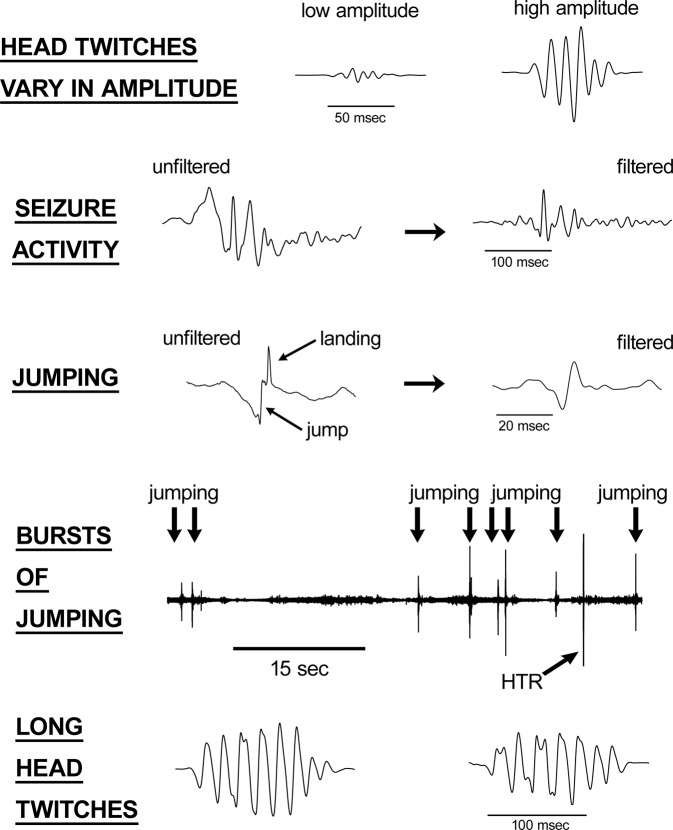
Plots of magnetometer voltage responses recorded from C57BL/6J mice. Filtering (40–200 Hz band-pass) was used as indicated. The recordings illustrate features of the head- twitch response (HTR) that can complicate analysis in the amplitude-time domain. First, the amplitude of the HTR waveform is highly variable, making it difficult to detect the behavior based on signal voltage. Second, other types of activity, such as seizures and jumping, can induce HTR-like waveforms, especially after the recordings are filtered to remove low-frequency head movement. Although hallucinogens do not normally induce escape behavior in rodents, some mice jump repeatedly after being placed in a magnetometer coil, potentially resulting in large numbers of false-positive detections. For example, part of a recording from a mouse that made at least 20 jumps during a 30-min test session is shown here. Third, head twitches induced by hallucinogens sometimes have anomalous features; most head twitches have a duration of <120 msec, but responses with a longer duration are sometimes observed.

Although use of a magnetometer increases the reliability and throughput of HTR studies, the recordings must be analyzed by manual assessment, which can be time-consuming. To further simplify data analysis, procedures have been developed to automatically detect the HTR in magnetometer recordings based on the specific features of the behavior^27,28^. The first published automated assessment method was based solely on signal voltage^27^. More recently, de la Fuente Revenga *et al*. detected the HTR based on the amplitude, frequency, and duration of head movement^28^. Unfortunately, those types of feature-based detections can be problematic because it is difficult to devise procedures that are sensitive to all HTRs. Detecting the HTR based on voltage is especially difficult because the amplitude of the response can vary over a wide-range (see Fig. 1). Indeed, ~20% of the responses recorded by Siegel *et al*. were low-amplitude and failed to trigger the detector^27^. Although de la Fuente Revenga *et al*. achieved better results using an approach based on multiple features, their procedures failed to detect 9.4% of the HTR in aged (~12 month-old) mice due to low amplitude^28^.

Another problem with feature-based detection methods is that other behaviors can induce HTR-like waveforms in the magnetometer recordings, potentially resulting in false-positive detections. As shown in Fig. 1, the waveforms associated with jumping and seizures match the amplitude, frequency and duration of the HTR, especially after the recorded signal is filtered to remove low-frequency activity. The HTR detection procedures developed by de le Fuente Revenga *et al*. could not distinguish between head twitches and jumping, necessitating the use of a piezo sensor to detect when mice jumped^28^. Although hallucinogens do not typically induce jumping in rodents, some mice make repeated escape attempts after being placed in a magnetometer coil, which would result in erroneous findings if not detected. An excerpt of a recording from a mouse that jumped repeatedly during a HTR experiment is shown in Fig. 1.

The existence of anomalous HTR must also be considered when developing procedures to automate data analysis. None of the HTR recorded in our initial series of experiments using a magnetometer had a duration exceeding 120 msec.^17^ However, further testing demonstrated that some HTR actually have a longer duration^19^. Fig. 1 shows two examples of HTR lasting ~150 msec. These types of anomalous responses only came to light after extended testing with multiple hallucinogens. Because the kinematics of head movement during the HTR can vary depending on the method of induction and the age and strain of the mice used in the experiments, automated detection methods should include procedures that can be used to verify that head twitches and other events in the recordings have been classified correctly.

Analysis of magnetometer recordings in the amplitude-time domain is not optimal because there is considerable variation in the amplitude of recorded HTR. Recent studies have used deep learning to successfully classify biometric signals by transforming the data into a visual representation in the time-frequency domain (a scalogram) using a wavelet transform^29–32^. Because the recorded signal is transformed into a visual representation, it can be analyzed using image classification techniques. For example, Smith and Kristensen used a Morlet wavelet to transform mouse ultrasonic vocalizations into scalograms and then used a convolutional neural network (CNN) to identify calls in the images^31^. Another group used continuous wavelet transform (CWT)-derived scalograms and a CNN to detect epileptic activity in EEG signals^30^. Similar approaches have been applied to the analysis of electrocardiogram (ECG) recordings and electromyographic (EMG) data^29,32^.

Because deep learning based on scalograms has been used to successfully classify other types of complex biometric signals, we investigated whether a similar approach can be used to automate HTR detection. According to recent studies, images containing time-frequency data can be very effectively analyzed using a multistage approach where features extracted using an off-the-shelf deep CNN are used to train a support vector machine (SVM) or another type of classifier^33–37^. Combining a pretrained deep CNN with an SVM often outperforms other architectures^33,34,38–40^. We have used this multistage approach to detect the HTR. After transforming the magnetometer data into scalograms using a wavelet transform, deep features were extracted using the existing 50-layer ResNet deep learning model and then the images were classified using an SVM. The 50-layer ResNet model is a deep CNN that is trained to classify 1,000 image types and includes residual learning, which improves performance by increasing the depth of representation^41^. There is considerable evidence that pretrained CNNs can be used to extract deep features from scalograms and other types of images that are distant from the original datasets used for training^33,34,38–40,42^. Features were extracted from a fully connected layer of ResNet-50 because deeper network layers generate a rich semantic image representation^42^ and are well suited for image recognition tasks^34,37,39^. Analysis of magnetometer recordings containing >10,000 HTR induced by hallucinogens confirmed that deep feature extraction combined with an SVM can be used to detect the behavior in a very sensitive manner, including low-amplitude and long-duration events. Furthermore, after transformation to scalograms, the HTR has a distinct appearance compared to jumping and seizures, so those events (and other types of head movement) generally do not trigger false-positive detections. An important feature of this analysis method is that images of the detected events are created automatically and can be used to evaluate the accuracy of the results, minimizing the risk of generating erroneous data.

## Results and discussion

### Generation of a dataset for training and validation

**Figure 2:**
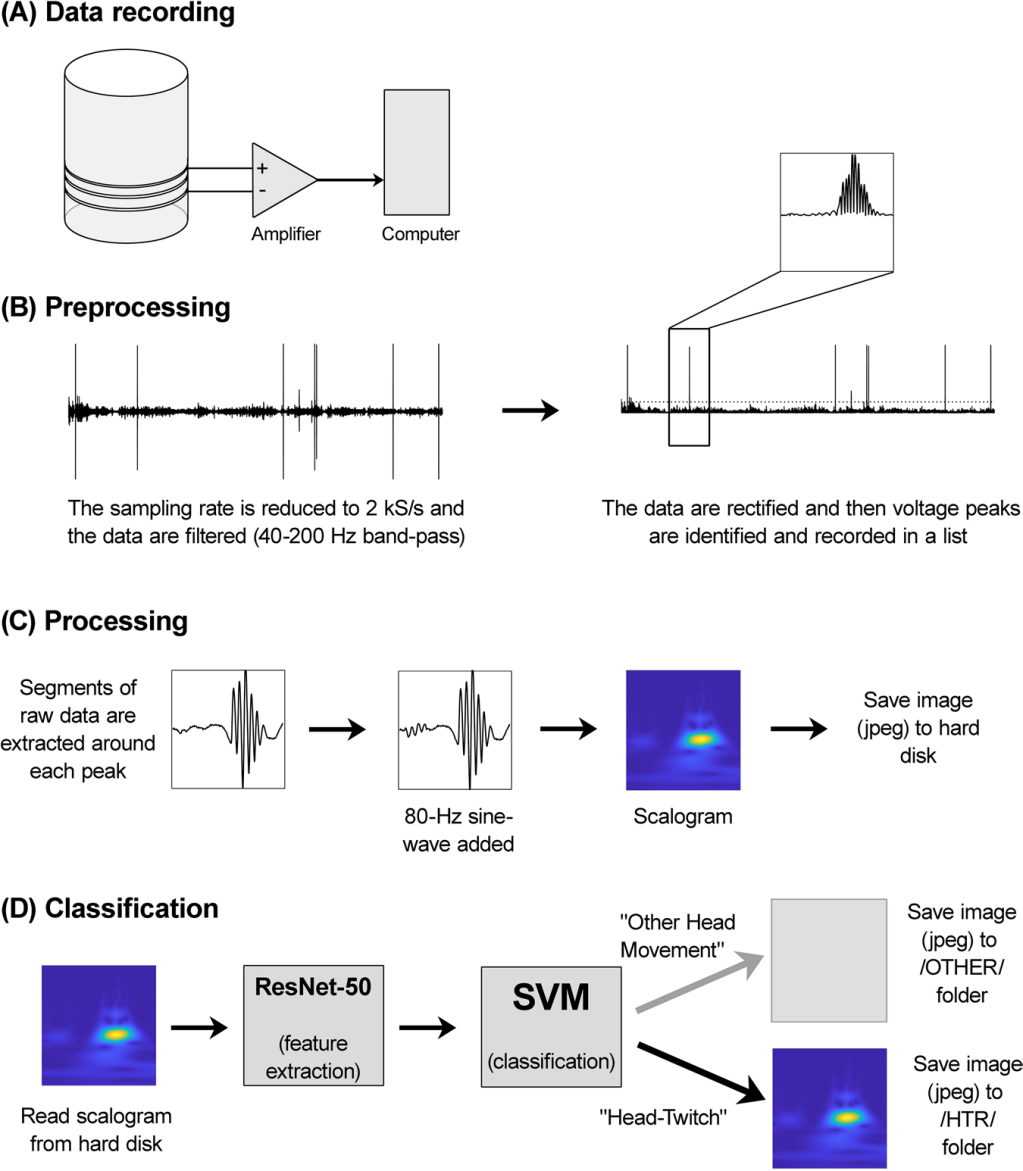
Summary of the procedures used to detect head twitches using scalograms and deep learning. (**A**) To record data during experiments, the voltage signal from the magnetometer coil is amplified, digitized, and saved to hard disk. (**B**) Events in the recordings are identified in the preprocessing step. During preprocessing, the recordings are re-sampled (2-kHz), filtered (40–200 Hz band-pass) and rectified, and then peaks exceeding a voltage threshold are identified. (**C**) Next, scalograms are generated during the processing step. Segments of data are extracted from the recordings, a brief 80-Hz sinusoidal signal is added, and then scalograms are created by continuous wavelet transform and saved to disk as image files. (**D**) Finally, the scalograms are classified. After the scalograms are read from disk, a Support Vector Machine (SVM) algorithm is used to classify the images based on features extracted by the deep convolutional neural network ResNet-50.

To create a dataset for training and validation, magnetometer recordings from published and unpublished experiments (*n* = 80 mice) were preprocessed as shown in Fig. 2B. In addition to HTRs, the recordings contained other types of activity that could potentially trigger false-positive detections (e.g., grooming, jumping, ambulation, seizures, and noise transients). The sampling rate of the data files was reduced to 2 kS/s and the recordings were filtered (40–200 Hz band-pass), rectified, and local maxima were identified using the *FindPeaks()* function from the MATLAB Signal Processing Toolbox. The *FindPeaks()* function searches for the tallest peak and then ignores all of the neighboring peaks within a specified distance. The same procedure is repeated for the next tallest peak, and so on, until all of the peaks exceeding a specified voltage threshold have been identified. Setting the minimum distance between peaks to 0.2 s and the voltage threshold to eight standard deviations above the mean rms value for each recording allowed HTRs to be identified without capturing excessive amounts of extraneous activity. Using a minimum peak-to-peak distance of 0.2 s is consistent with published procedures used to detect the HTR^28^.

Figure 2C summarizes the subsequent data processing steps. Once all of the peaks were identified, short segments of the unfiltered recordings were extracted for further analysis. Each extracted segment contained 441 samples, with 280 samples before the peak, and 160 samples after the peak. A subthreshold 80-Hz sinusoidal signal (89 samples long, amplitude 50% of the preprocessing threshold) was inserted at the beginning of each segment. Scalograms show the relative energy density of a signal, so low-level sinusoidal noise is more likely to be misclassified as a HTR if the ongoing level of activity in an extracted segment is relatively low. The inserted reference sinusoidal signal becomes visible in the scalograms when the system is operating in a region where subthreshold noise is more likely to be misclassified, potentially serving as a feature that is extracted by the CNN. Finally, the segments were transformed into scalograms using the continuous wavelet transform (CWT). The wavelet transform was applied to the data using a Morse wavelet with signal length, sampling frequency, and voices per octave of 441, 2000, and 12, respectively. Each scalogram was saved to disk as an RGB jpeg file with a 227 × 227-pixel resolution.

### Training a support vector machine to classify head twitches

The processing step yielded a total of 4,673 scalograms. The scalograms containing head twitches (“HTR” category, *n* = 3,370) or other types of activity (“OTHER” category, *n* = 1,303) were identified by examination of the corresponding magnetometer data using published procedures^19^. After constructing a database containing 1,303 scalograms from each category, the images were randomly divided into training and test sets, with a 1:3 training:test ratio. Next, ResNet-50 was used as a feature extractor to train a multiclass SVM image category classifier to perform a one-versus-all classification of the scalograms^41,43^. The scalograms were resized to 224 × 224 pixels to match the input dimensions of ResNet-50. Features were extracted from layer “fc1000” of ResNet-50 and used to train the SVM using a fast stochastic gradient descent solver. For the test set, the trained classifier had an accuracy of 98.87% for both the “HTR” and “OTHER” categories (i.e., out of 977 images from each category, 966 were correctly classified). The trained classifier was then saved as an error-correcting output coding (ECOC) algorithm^44^.

### Validation of the procedures used to automate HTR detection

**Figure 3:**
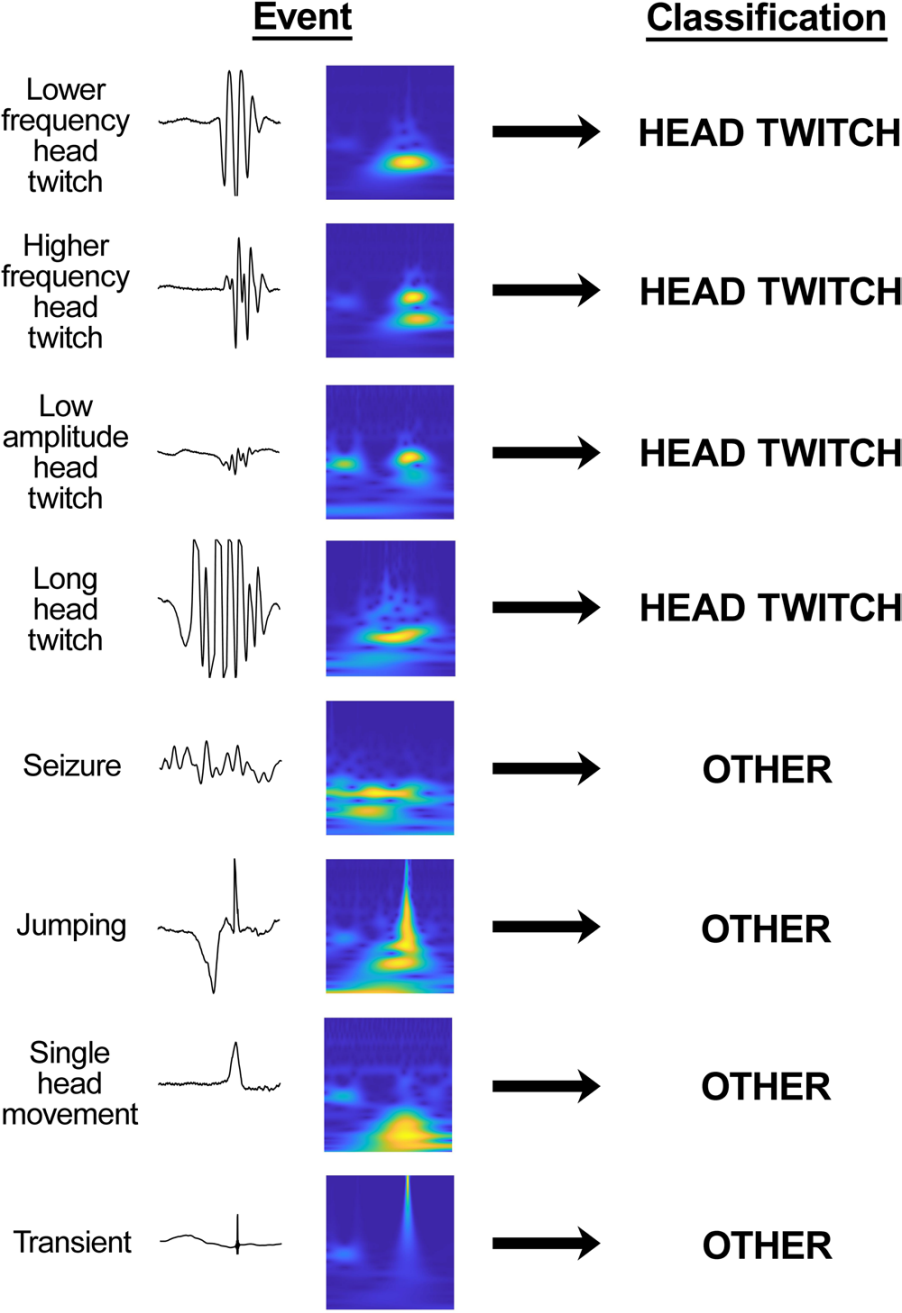
Representative examples of classifications made using scalograms and deep learning. Several different types of events that were recorded from C57BL/6J mice are shown before and after transformation to scalograms. The predictions made by the support vector machine are shown on the right side.

Although the automated detection procedures were sensitive to HTR in the test set and could distinguish them from other types of activity, further studies were necessary to fully validate the technique. We have conducted hundreds of HTR experiments with hallucinogens, providing a large dataset that can be used to test the detection procedures. Data files from 237 mice were re-analyzed using the procedures shown in Fig. 2B–D. A variety of hallucinogens were tested in the experiments, including LSD, psilocybin, mescaline, *R*-(–)-4-iodo-2,5-dimethoxyamphetamine (DOI), 4-bromo-2,5-dimethoxyamphetamine (DOB), and *N*-(2-methoxybenzyl)-2,5-dimethoxy-4-iodophenethylamine (25I-NBOMe). The results of the analysis are summarized in Table 1. A total of 11,312 HTR were identified in the recordings using published analysis procedures^19^. As shown in Table 1, the multistage CNN-SVM approach detected 11,193 of the HTR (98.95%). The percentage of HTR detected in each set of recordings ranged from 97.03% to 99.74%, with a mean ± SEM detection rate of 99.02 ± 0.28%. As shown in Fig. 3, the SVM was able to detect HTR that have a relatively long duration (i.e., 0.12–0.16 s). HTR with low amplitude were also detected (Fig. 3). Although the amplitude of the HTR reportedly declines in older mice^28^, detection accuracy was not affected by the age of the animals used in the experiments (*R* = −0.2217, *n* = 11, *p* = 0.5125).

**Table 1:** Results of the extended validation performed using 237 recordings from mice.

Experiment	Drug	Age (weeks)	Duration (min)	*N*	Manual HTR counts	Automated HTR counts	Percent detected^a^	False positive detections	Total errors^b^	Total error rate^c^	*R* value
1	LSD	23	30	24	1,311	1,300	99.16%	1	12	0.92%	0.9994
2	25I-NBOMe	20	30	25	1,472	1,466	99.59%	10	16	1.09%	0.9989
3	2C-I	30	30	27	1,388	1,378	99.28%	3	13	0.94%	0.9997
4	25I-NBMD	45	20	13	297	289	97.31%	0	8	2.69%	0.9986
5	1-Butanoyl-LSD	18	30	31	975	972	99.69%	15	18	1.85%	0.9987
6	DOB-FLY	36	30	23	1,771	1,755	99.10%	6	22	1.24%	0.9997
7	*R*-(–)-DOI	34	30	8	633	629	99.37%	2	6	0.95%	0.9997
8	Mescaline	37	30	27	855	852	99.65%	2	5	0.58%	0.9999
9	DOB	20	30	33	1,819	1,765	97.03%	14	68	3.74%	0.9984
10	Psilocybin	30	30	16	403	400	99.26%	5	8	1.99%	0.9990
11	2C-T-7	25	30	10	388	387	99.74%	3	4	1.03%	0.9997
Totals:				237	11,312	11,193	98.95%	61	180	1.59%	

^a^ Percent detected = (automated HTR count ÷ manual HTR count) × 100.

^b^Total errors = number of false positive detections + number of head twitches that were detected manually but not by the automated procedures.

^c^Total error rate = total errors ÷ manual HTR count.

In addition to the HTR, many other types of head movement were accurately classified by the SVM (see Fig. 3). Inspection of the results showed that jumping, single head movements, and noise transients did not trigger false-positive detections. For example, one of the mice shown in Fig. 1 made 20 identifiable jumps during a 30-min test session; all of those escape attempts were classified in the “OTHER” category by the SVM. Although there were a few false-positive detections (*n* = 61), most of the false-positives occurred when mice made two high-frequency head movements in rapid succession. Although we do not classify events containing <three head movements as HTR in our studies^17,19^, the decision to exclude those events was made to increase the reliability of our assessment procedures and is not based on a formalized definition of the behavior. HTR involving only two sequential head movements were not observed in high-speed video recordings of mice treated with (±)-DOI^17^. However, there may be instances where the magnetometer responds weakly to head rotations in one direction.

In summary, using scalograms and deep learning to detect the HTR closely mirrors the results obtained using established assessment procedures. There is a robust correlation (*R* = 0.9992, *n* = 237, *p* < 0.0001) between the number of HTR detected using scalograms and deep learning vs. existing manual assessment methods^17,19^. The total error rate, including both HTR detection failures and false-positive detections, was 1.59% (Table 1). The mean ± SEM error rate across the 11 experiments was 1.55 ± 0.29%.

### Optimization of the preprocessing procedures

Almost all of the missed detections in Table 1 reflect preprocessing errors rather than misclassifications made by the SVM. Typically, the missed detections occurred in recordings with high levels of baseline activity within the 40–200 Hz frequency band, resulting in relatively high thresholds for the *FindPeaks()* function (e.g., 15–25% of the voltage ceiling for the recording). Reducing the preprocessing threshold globally would likely increase the proportion of HTRs that are identified during the preprocessing step, but that option was rejected because the current threshold is appropriate for most of the recordings (setting the threshold too low can have detrimental effects on computational load and false-positive detections). As an alternative, the *FindPeaks()* threshold can be constrained so that it does not exceed a ceiling value. Setting the *FindPeaks()* threshold to ≤10% of the maximum voltage for the recordings increased the proportion of head twitches that were detected but also increased the false-positive rate (data not shown). Conversely, as shown in Table 2, constraining the threshold to ≤15% of the maximum voltage increased the detection accuracy without significantly altering the number of false-positive detections. Using the latter procedure, the SVM detected 99.40% of the HTR in the recordings (11,244 out of 11,312 HTR were identified and correctly classified). Including false-positive detections (*n* = 80), the total error rate was 1.31%.

**Table 2:** Performance of the automated head-twitch response (HTR) detection after optimization of the preprocessing procedures.

Experiment	Drug	Age (weeks)	Duration (min)	*N*	Manual HTR counts	Automated HTR counts	Percent detected^a^	False positive detections	Total errors^b^	Total error rate^c^	*R* value
1	LSD	23	30	24	1,311	1,304	99.47%	2	9	0.69%	0.9995
2	25I-NBOMe	20	30	25	1,472	1,472	100.00%	12	12	0.82%	0.9994
3	2C-I	30	30	27	1,388	1,381	99.50%	5	12	0.86%	0.9998
4	25I-NBMD	45	20	13	297	292	98.32%	0	5	1.68%	0.9988
5	1-Butanoyl-LSD	18	30	31	975	972	99.69%	15	18	1.85%	0.9987
6	DOB-FLY	36	30	23	1,771	1,763	99.55%	14	22	1.24%	0.9997
7	*R*-(–)-DOI	34	30	8	633	629	99.37%	2	6	0.95%	0.9997
8	Mescaline	37	30	27	855	852	99.65%	2	5	0.58%	0.9999
9	DOB	20	30	33	1,819	1,791	98.46%	17	45	2.47%	0.9991
10	Psilocybin	30	30	16	403	401	99.50%	6	8	1.99%	0.9991
11	2C-T-7	25	30	10	388	387	99.74%	5	6	1.55%	0.9994
Totals:				237	11,312	11,244	99.40%	80	148	1.31%	

^a^ Percent detected = (automated HTR count ÷ manual HTR count) × 100.

^b^Total errors = number of false positive detections + number of head twitches that were detected manually but not by the automated procedures.

^c^Total error rate = total errors ÷ manual HTR count.

Although the amplitude of the head movement during a HTR typically increases and then declines symmetrically over the course of the response, some HTR may be more asymmetrical. Therefore, the fact that the position of the HTR in the scalograms is not fixed and depends on the location of the local voltage maxima within the response is a potential source of error. To address this potential confound, we examined whether the detection accuracy is altered by shifting the location of the detected peaks (see Table 3). The analysis was performed on a subset of the mice (*n* = 10) from Table 2. Shifting all of the detected peaks forward in time (i.e, adding a given amount of time to each peak location) by up to 40 msec had no effect on detection accuracy. Likewise, shifting all of the detected peaks backward in time by up to 20 msec also had no effect on accuracy. Larger backward shifts reduced accuracy (a 30-msec backward shift reduced accuracy from 99.22% to 93.73%, whereas a 40-msec backward shift reduced accuracy to 75.72%) because some of the HTRs were partially shifted out of the frame of the scalogram (effectively reducing the duration of those HTR). The latter phenomenon did not occur spontaneously in any of the recordings analyzed during the validation studies. Furthermore, considering the fact that most HTR have a duration of ~40–60 msec, these results confirm that the analysis procedures can tolerate a considerable degree of variability in the location of the detected local maxima.

**Table 3:** Effect of variability in the location of detected peaks on the accuracy of the head-twitch response (HTR) analysis procedures.

Time shift (msec)	Proportion of head twitches detected	Accuracy
−40	290/383	75.72%
−30	359/383	93.73%
−20	376/383	98.17%
−10	379/383	98.96%
0	380/383	99.22%
+10	380/383	99.22%
+20	380/383	99.22%
+30	379/383	98.96%
+40	378/383	98.69%

### Comparison of the results obtained using automated HTR detection vs. traditional video scoring

To further test the accuracy of the automated HTR detection procedures, scalograms and deep learning were used to re-analyze an experiment where the response to (±)-DOI was assessed using simultaneous video and magnetometer recordings^17^. In the experiment, (±)-DOI (0, 0.25, 0.5, and 1 mg/kg IP) was administered to 13 mice using a within-subjects design. Out of the 753 HTR identified in the video recordings, 745 (98.94%) were detected by automated analysis of the magnetometer data. There were two false-positive automated detections, yielding a total error rate of 1.33%. Two additional HTR that were detected in the magnetometer data could not be positively identified in the video recordings, likely because the frame rate (30 fps) was too low to fully capture the behavior. The HTR counts generated using the two methods are highly correlated (*R* = 0.9992, *n* = 52, *p* < 0.0001). These results are consistent with the performance in Table 2.

### Evaluating the specificity of the automated HTR detection procedures

Additional analyses were conducted to confirm that the automated HTR detection procedures are not triggered by other types of head movement. Because jumping can potentially trigger false-positive detections in automated HTR assessments^28^, we examined whether the current approach can reliably distinguish between jumping and the HTR. To induce jumping, pellets containing 75 mg of morphine were implanted subcutaneously in two mice and naloxone (1 mg/kg IP) was injected 5 days later. These procedures induce jumping and other abstinence signs in mice^45^. Out of 107 jumps made during the 10-min assessment period, two triggered false-positive HTR detections (a false-discovery rate of 1.9%).

To determine whether high levels of grooming and other types of motor activity can trigger false-positive detections, published experiments^17^ with (+)-amphetamine and the dopamine D_1_ receptor agonist SKF38393 were re-analyzed. As was the case for the experiment with (±)-DOI, simultaneous video and magnetometer recordings were available for the experiments with (+)-amphetamine and SKF38393. Although we found previously that 10 mg/kg SKF38393 increased the duration of grooming in the experiment^17^, the grooming behavior did not trigger any false-positive detections: 36 “HTR” and 215 “OTHER” events were detected during the preprocessing step, all of which were classified correctly by the SVM. In the second experiment, where mice were treated with 0, 2.5 and 5 mg/kg (+)-amphetamine, 675 events were detected during preprocessing (22 “HTR” and 653 “OTHER” events); all of the detected events were classified correctly by the SVM.

Sensitivity to seizures was also examined. Although seizures do not normally occur in studies with hallucinogens, it is important to confirm that HTR detections are not triggered by convulsive head movements. Treatment with certain D_1_ receptor agonists can induce seizure activity in rodents^46^. Convulsive movements were observed in two mice treated with the dopamine D_1/5_ receptor agonist SKF-82958 (3 mg/kg IP). Of the 103 events detected in the recordings, 102 were classified correctly (1 “HTR” and 101 “OTHER” events). The one event that was misclassified as a HTR (a false-positive rate of 0.97%) was triggered by two high-frequency head movements made in rapid succession (the same phenomenon that was discussed earlier) and was not coincident with seizure activity.

### Procedures to evaluate the performance of automated HTR detection systems

**Figure 4:**
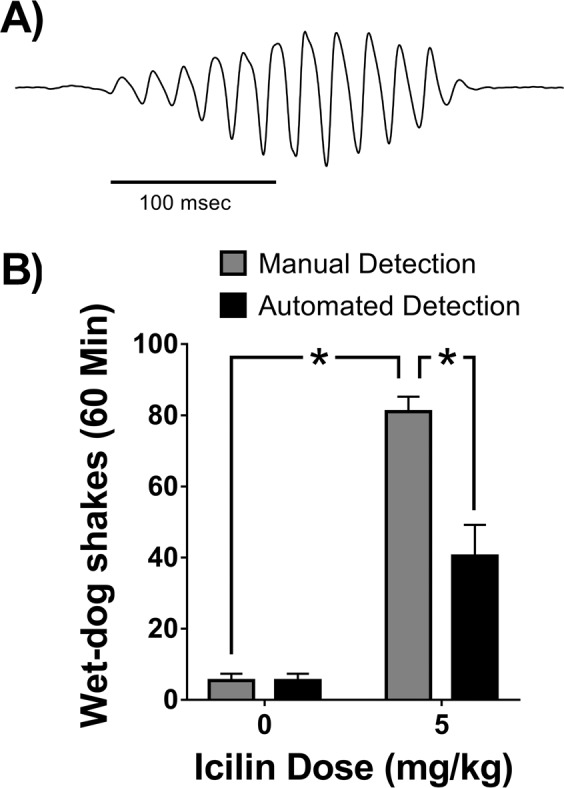
Wet-dog shakes induced by icilin, a transient receptor potential M8 (TRPM8) agonist, in C57BL/6J mice. (**A**) An unfiltered magnetometer voltage trace showing a wet-dog shake induced by icilin. As shown here, many of the wet-dog shakes induced by icilin have a duration exceeding 200 msec. (**B**) Only 50% of the wet-dog shakes induced by icilin were identified as head-twitches by the CNN-SVM. The data are presented as group means ± SEM. Each group contained *n* = 5 mice. *p < 0.001, significant difference (Tukey’s test).

**Figure 5:**
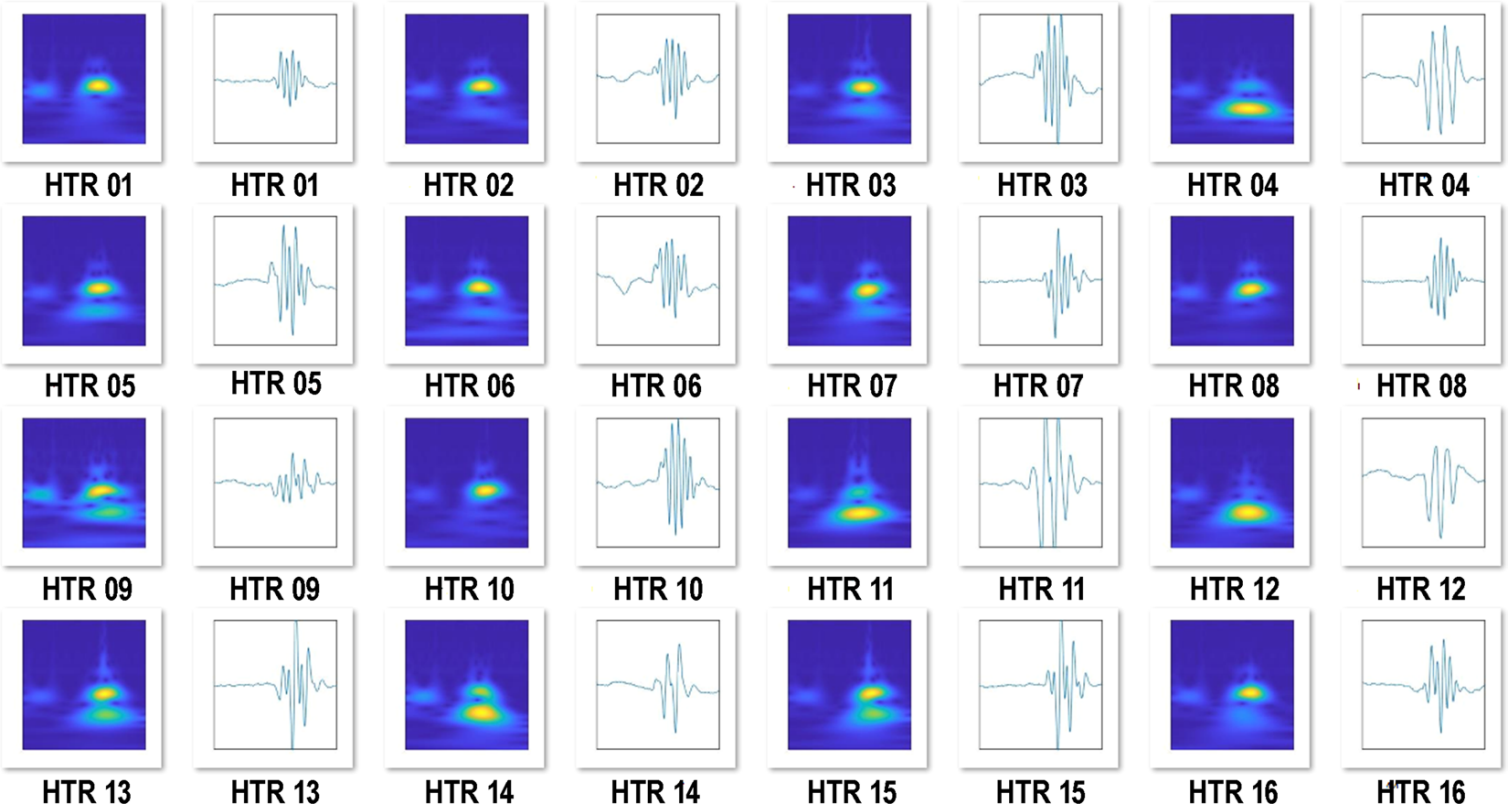
An important feature of the analysis method is that images of the detected events are created automatically. After a recording is analyzed, the scalograms and images of the associated waveforms are saved in one of two folders depending on how they were classified. The image files can be reviewed to detect anomalous events and classification errors.

It is important to recognize that all automated HTR analysis methods will have limitations. As shown in Fig. 1, there is considerable variability in the amplitude and duration of the head movement during the HTR. Because of this variability, it should not be assumed that automated HTR analysis techniques can detect all responses with exactly the same sensitivity and reliability. Furthermore, the HTR is not the only type of shaking behavior that can be induced by pharmacological agents. The transient receptor potential cation-channel M8 (TRPM8) agonist icilin^47^ induces wet-dog shakes in a variety of species, including mice^48^. As shown in Fig. 4A, the duration of shaking induced by icilin often exceeds 200 msec. Because wet-dog shakes induced by icilin have a longer duration compared to HTRs, only 50% of the responses induced by icilin (5 mg/kg IP) in male C57BL/6J mice were classified as HTR by the SVM (Fig. 4B). Unfortunately, without carefully inspecting the data, there would have been no way to know that the automated detection procedures had failed with icilin. To minimize the risk of generating erroneous data, fully automated HTR detection systems should include procedures so that their performance can be evaluated when they are used to assess shaking behavior induced by new pharmacological manipulations. One benefit of using scalograms to analyze magnetometer recordings is that images of the detected events are created automatically. Specifically, when a recording is analyzed using the procedures in Fig. 2, the scalograms and images of the associated waveforms are saved in one of two folders depending on how they were classified. As shown in Fig. 5, the images can be examined to detect anomalous events and classification errors.

## Conclusions

Deep learning based on scalograms can be used to detect the HTR, allowing the behavior to be assessed in a fully-automated manner with extremely high sensitivity and selectivity. Analysis of HTRs as one-dimensional (1D) representations can potentially fail due to the variability of their features in the amplitude-time domain and potential overlap with other behaviors; after transformation into two-dimensional (2D) time-frequency representations, however, HTR can be unambiguously identified and reliably discriminated from other types of head movement. The fact that a 2D representation of the HTR performs better than a 1D representation is not surprising. Although transforming the recordings into scalograms eliminates information about signal amplitude, it actually provides a richer representation of the recorded events. In additional to providing a multidimensional (2D) representation, the scalograms also incorporate the activity that occurred before and after each HTR, information that can potentially facilitate classification.

Impressive results were obtained with these analysis procedures. It should be noted, however, that these procedures have only been validated for C57BL/6J mice and the 11 compounds shown in Tables 1 and 2. Although it is anticipated that the analysis procedures will show similar performance when applied to other drugs, instruments, and strains of mice, it is possible that the analysis procedures may have to be adjusted when they are applied to other test conditions. Although the SVM did not detect all of the responses induced by icilin, sensitivity to wet-dog shakes can probably be augmented by increasing the number of samples included in each scalogram. Future studies will investigate whether the present approach can also be used to detect wet-dog shakes and other types of behaviors in magnetometer recordings.

In the present investigation, scalograms and deep learning were used to analyze magnetometer recordings generated using magnets attached to the cranium. However, this analysis approach is likely to be equally effective with other types of magnetometer recordings (e.g., experiments performed using magnetic ear tags^49^ or magnets glued to the scalp^27^). All of the available magnet attachment methods have advantages and limitations, and are not necessarily interchangeable. The latter two methods do not require surgeries, which is convenient for acute or short-term studies. On the other hand, mice with magnet implants can be tested repeatedly over an extended time period (cohorts of mice can be maintained for 5–6 months, which is considerably longer than is possible with other approaches). In addition, magnets attached to the cranium with dental cement are less likely to be removed or dislodged by cagemates, facilitating group housing. Single housing influences 5-HT_2A_ receptor expression^50–52^ and can dramatically alter the magnitude of the HTR^53–55^.

In addition to the present technique, several other methods have been developed to automatically detect HTR behavior in magnetometer recordings. Siegel *et al*.^27^ used a very rudimentary approach where all of the recorded events exceeding a criterion amplitude were classified as HTR, resulting in a false-negative rate of ~20%. de la Fuente Revenga *et al*. achieved better results by detecting the HTR based in amplitude, frequency, and duration^28^. Nevertheless, although the sensitivity of their approach was high in young mice (the procedures detected 98.61% of the HTR induced by DOI in 10-week-old mice), performance was lower in older animals. Jumping also triggered false-positive HTR detections, necessitating the use of an additional piece of hardware to detect when mice jumped. More recently, the same group published an updated HTR analysis method that includes an additional spectral analysis step, allowing HTR and jumping to be discriminated unambiguously^49^. The increased selectivity of the technique was offset by a reduction in sensitivity — 96.83% of the HTR were detected in a validation experiment performed in young mice. Although that level of performance is acceptable in young animals, if the sensitivity of the technique declines over time as mice age then the false-negative rate could be considerably higher in some experiments.

Although these studies were conducted to test the hypothesis that a multistage CNN-SVM approach based on scalograms can be used to detect the HTR, another goal was to develop an automated analysis method that has the same sensitivity and selectivity as manual scoring. Validation studies have confirmed that manual analysis procedures are extremely sensitive to HTR behavior^17,19^. In addition, manual analysis is highly insensitive to false-positive detections and is unlikely to be confounded by HTR with anomalous features. The present results confirm that scalograms and deep learning can be used to detect the HTR with the same sensitivity and selectivity as existing manual assessment methods.

Finally, in addition to having high sensitivity and reliability, another advantage of the present technique is the availability of image files showing all of the detected events; the images can be reviewed to evaluate the performance of the analysis procedures. Indeed, the relatively small number of false-positive detections in Tables 1 and 2 were easily identified by examining the image files. Using a magnetometer coil to assess head movement can markedly increase the accuracy of the HTR assay. The present procedures can be used to automate the detection of HTR in magnetometer recordings without any apparent loss of sensitivity or reliability.

## Methods

### Analysis of magnetometer recordings from previous experiments

With the exception of studies with icilin and morphine (see below), existing magnetometer recordings were re-analyzed in these studies. The experiments with *d*-lysergic acid diethylamide (LSD), psilocybin, 2,5-dimethoxy-4-iodophenethylamine (2C-I), *N*-(2-methoxybenzyl)−2,5-dimethoxy-4-iodophenethylamine (25I-NBOMe), and *N*-(2,3-methylenedioxybenzyl)−2,5-dimethoxy-4-iodophenethylamine (25I-NBMD), 1-butanoyl-LSD (1B-LSD), mescaline, 4-bromo-2,5-dimethoxyamphetamine (DOB), (±)−4-iodo-2,5-dimethoxyamphetamine (DOI), and 8-bromo-2,3,6,7-tetrahydro-α-methyl-benzo[1,2-*b*:4,5-*b’*]difuran-4-ethanamine (DOB-FLY) were included in previous publications^17,19,21,22,24,56^. The experiments with 2,5-dimethoxy-4-propylthiophenethylamine (2C-T-7; Cayman Chemical, Ann Arbor, MI, USA) and *R*-(–)-DOI (National Institute on Drug Abuse, Rockville, MD, USA) are unpublished. Male C57BL/6J mice from Jackson Laboratories (Bar Harbor, ME, USA) were used in all of the experiments. The data files were created using the procedures in Fig. 2A. Briefly, data were recorded a PowerLab 8SP data acquisition system (ADInstruments Inc., Colorado Springs, CO, USA). Magnetometer coil voltage was amplified, filtered to remove frequencies above 2–10 kHz, digitized (20 or 40 kS/s sampling rate, 16-bit ADC), and saved to disk in LabChart (*.adicht) format. Head twitches were identified in the recordings using published procedures^19^. All animal experiments were carried out in accordance with National Institute of Health guidelines and were approved by the University of California San Diego Institutional Animal Care and Use Committee.

### Detection of the head-twitch response using scalograms and deep learning

Analysis of magnetometer recordings using scalograms and deep learning was performed using MATLAB release 2019b (The MathWorks Inc., Natick, Massachusetts, USA). The recordings were converted to WAVE (*.wav) format prior to analysis. The MATLAB script used to detect and classify head twitches is included as a supplemental file. A second supplemental file includes a MATLAB script that can be used to train an SVM and save it as an ECOC algorithm, which is required to perform the analysis. Linear regression and statistical analyses were performed using Prism 7.00 (GraphPad Software, San Diego, CA, USA).

### Studies with icilin and morphine

Male C57BL/6J mice were housed in a vivarium at the University of California San Diego. Mice were housed up to four per cage in a climate-controlled room on a reverse-light cycle (lights on at 1900 h, off at 0700 h). Food and water were available *ad libitum* except during behavioral testing, which was conducted between 1000 and 1800 h. The animal facility meets all federal and state requirements for care and treatment of laboratory animals and is approved by the Association for Assessment and Accreditation of Laboratory Animal Care (AAALAC).

Mice were anesthetized using ketamine (100 mg/kg IP) and acepromazine (5 mg/kg IP). A neodymium magnet (4.57 mm × 4.57 mm × 2.03 mm, 375 mg) was attached to the cranium with dental resin. Experiments were conducted after a suitable recovery period (>7 days).

Icilin. Icilin (Cayman Chemical, Ann Arbor, MI, USA) was dissolved in water containing 17% Tween-80 and injected IP at a volume of 10 mL/kg. Immediately after injection, head movement was recorded in a glass cylinder surrounded by a magnetometer coil^19^ for 60 min.

Morphine. Two mice were subcutaneously implanted with 75 mg morphine pellets (National Institute on Drug Abuse, Rockville, MD, USA). Five days later, naloxone (1 mg/kg) was injected IP, and head movement was recorded in a magnetometer coil chamber for 10 min. Videos of the mice were also recorded to capture evidence of jumping behavior.

## Supplementary information

Supplementary Information.

Supplementary Information.

## Data Availability

The datasets generated during the current study are available from the corresponding article on reasonable request.
