# Supplementary material for: Automated detection of the head-twitch response using wavelet scalograms and a deep convolutional neural network
Source: Sci Rep. 2020 May 20;10:8344. doi: 10.1038/s41598-020-65264-x (PMC7239849; doi:10.1038/s41598-020-65264-x)
Supplement: Supplementary file 1 — Supplementary Information. [file 41598_2020_65264_MOESM1_ESM.docx]

Automated detection of the head-twitch response using wavelet scalograms and a deep convolutional neural network

Adam L. Halberstadt^1,2*^

*^1^ Department of Psychiatry, University of California San Diego, La Jolla, CA, USA*

*^2^ Research Service, VA San Diego Healthcare System, San Diego, CA, USA*

% HTR classification program

% created by Dr. Adam L. Halberstadt (ahalberstadt [at] ucsd.edu)

% version 10 (March 1, 2020)

% Use this program to identify events in magnetometer coil recordings

% and then predict whether they are head twitches.

% This program is a MATLAB Live Code File. Execution in MATLAB requires the

% following toolboxes: Deep Learning Toolbox, Wavelet Toolbox,

% Signal Processing Toolbox, Statistics and Machine Learning Toolbox, Image

% Processing Toolbox, and Deep Learning Toolbox Model for ResNet-50 Network.

% Use this file to create a *.mlx Live Code File in MATLAB.

% This program requires a Support Vector Machine (SVM), which is saved as an

% error-correcting output coding (ECOC) file in the same directory as the

% *.mlx file.

% If using an ECOC file with a different name, then edit the appropriate line below.

% The current ECOC is called "HTR.mat"

% Differences between magnetometer systems may require fine-tuning the

% preprocessing parameters (eg, the *minht* variable) and generating a new ECOC file.

% Instructions for completing those tasks are provided in a seperate file.

% Data files for analysis must be saved as WAVE files. When recording magnetometer

% coil data, the gain should be set so that the amplitude of the head twitches

% in the recordings is close to the ceiling for the ADC, but with only a small amount

% of clipping. If the gain is too low then it may be more difficult to detect low-amplitude

% responses. But if the amplitude is too high then background noise may interfere with

% HTR detection (the performance of both manual and automated analysis methods is

% adversely affected if the amplification level is too low or too high).

[file,path,indx] = uigetfile({'*.wav'}, 'File Selector');

if isequal(file,0)

disp('User selected Cancel')

else

disp(['User selected ', fullfile(path, file)])

end

prompt = {'Enter mouse ID:','Enter analysis duration: '};

dlgtitle = 'Input';

dims = [1 35];

definput = {'','30'};

answer3 = inputdlg(prompt,dlgtitle,dims,definput);

msID = answer3{1};

answer3b = str2num(answer3{2});

fullname = fullfile(path,file);

[filepath,name,ext] = fileparts(fullname);

[data,fs] = audioread(fullname);

if (fs > 2000)

data = resample(data,2000,fs);

disp('Resampling data file to 2 KHz sampling rate.')

else

disp('Sampling rate = 2 KHz.')

end

folderjpg = fullfile(filepath,msID,'jpg',filesep);

folderwave = fullfile(filepath,msID,'wave',filesep);

folderHTR = fullfile(filepath,msID,'HTR',filesep);

folderOTHER = fullfile(filepath,msID,'OTHER',filesep);

folderbase = fullfile(filepath,msID,filesep);

mkdir(folderjpg);

mkdir(folderwave);

fs = 2000;

filebytes = size(data);

fileleng = floor((filebytes(1,1))/fs/60);

if answer3b > fileleng

last = fs*60*fileleng;

else

last = fs*60*answer3b;

end

data = data(1:last, :); % keep the data selected for analysis

% preprocessing step

dataFilt = bandpass(data,[40 200],fs,'Steepness',0.8);

dataabs = abs(dataFilt);

datasd = std(dataabs);

mean = rms(dataabs);

minht = mean + (datasd*8); % minht sets the peak detection threshold

if minht > 0.15

minht = 0.15;

else

minht = minht;

end

[pks,locs] = findpeaks(dataabs,fs,'MinPeakDistance',0.2,'MinPeakHeight',minht);

% processing step -- go through the peaks one by one and transform the data into images

d = size(locs);

for k = 1:1:d

curpos = locs(k);

if (curpos < 0.06) % check to see if this is the begining of the file

a = curpos;

b = curpos + 0.22;

elseif ((curpos*fs) >= (last-(0.08*fs))) % check to see if this is the end of the file

j = (last-curpos*fs)/fs;

a = curpos - 0.22 + j;

b = curpos + j;

else

a = curpos - 0.14;

b = curpos + 0.08;

end

% read the segment

h = floor(a*fs);

i = floor(b*fs);

segment = data(h:i, :);

% add an 80-Hz sinusoidal waveform to the beginning of the segment

f = 80;

ts = 1/2000;

T = 0.05;

t = 0:ts:T;

segment_sine = (minht/2)*sin(2*pi*f*t);

time_seg = length(segment);

segment_start = zeros(1,20);

segment_start2 = horzcat(segment_start,segment_sine);

segment_start2(110:time_seg) = 0;

segment_sine_reshape = reshape(segment_start2,[],1);

segment2 = segment + segment_sine_reshape;

% perform the continuous wavelet transform

fb = cwtfilterbank('SignalLength',time_seg,...

'SamplingFrequency',fs,...

'VoicesPerOctave',12);

[cfs,frq] = wt(fb,segment2);

% create and save the scalogram

time = length(segment2)./fs;

t = 0:time/(length(segment2)):time-time/length(segment2);

fig = pcolor(t,frq,abs(cfs));

ylim([-1 1]);

set(gca,'XTickLabel',[],'YTickLabel',[]);

set(gca,'yscale','log');shading interp;axis tight;

set(gcf,'Position', [100, 100, 145, 145]);

filename2 = [num2str(name), num2str(curpos*fs), '.jpg'];

fullname3 = fullfile(folderjpg,filename2);

saveas(fig,fullname3,'jpeg');

% create and save an image of the signal waveform for comparison

time = length(segment)./fs;

t2 = 0:time/(length(segment)):time-time/length(segment);

fig2 = plot(t2,segment);

ylim([-1 1]);

set(gca,'XTick',[],'YTick',[]);

set(gcf,'Position', [100, 100, 145, 145]);

filename4 = [num2str(name), num2str(curpos*fs), '.jpg'];

fullname5 = fullfile(folderwave,filename4);

saveas(fig2,fullname5,'jpeg');

end

mkdir(folderHTR);

mkdir(folderOTHER);

% load the scalograms for analysis

imds = imageDatastore(folderjpg);

% load the CNN

net = resnet50();

featureLayer = 'fc1000';

imageSize = net.Layers(1).InputSize;

% load the SVM

matfile = fullfile(filepath,'\HTR.mat');

classifier2 = load(matfile);

% figure out how many scalograms need to be classified

a = dir([folderjpg '/*.jpg']);

x = numel(a);

fileid = fopen( 'results.txt', 'wt' );

fprintf( fileid, 'Subject %s\n', msID);

for b = 1:x

% Get one of the scalograms from the image data store for analysis

[testImage,info] = readimage(imds,b);

% Resize the scalogram to match the input layer of the CNN

ds = augmentedImageDatastore(imageSize, testImage,'ColorPreprocessing', 'gray2rgb');

% Extract the features of the scalogram using the CNN

imageFeatures = activations(net, ds, featureLayer, 'OutputAs', 'columns');

% Pass the features to the SVM and make a prediction

prediction = predict(classifier2.classifier, imageFeatures, 'ObservationsIn', 'columns');

fprintf( fileid, '%s,%s,%s\n', num2str(b), info.Filename, prediction);

[filepath2,name2,ext2] = fileparts(info.Filename);

fullname6 = fullfile(folderwave,name2);

fullname6 = [fullname6, '.jpg'];

fullname7 = fullfile(folderHTR,name2);

fullname7 = [fullname7, 'w.jpg'];

fullname8 = fullfile(folderOTHER,name2);

fullname8 = [fullname8, 'w.jpg'];

if prediction == 'HTR'

copyfile(info.Filename,folderHTR);

copyfile(fullname6,fullname7);

else

copyfile(info.Filename,folderOTHER);

copyfile(fullname6,fullname8);

end

end

fclose(fileid);

copyfile('results.txt',folderbase);
