# Supplementary material for: Automated detection of the head-twitch response using wavelet scalograms and a deep convolutional neural network
Source: Sci Rep. 2020 May 20;10:8344. doi: 10.1038/s41598-020-65264-x (PMC7239849; doi:10.1038/s41598-020-65264-x)
Supplement: Supplementary file 2 — Supplementary Information. [file 41598_2020_65264_MOESM2_ESM.docx]

Automated detection of the head-twitch response using wavelet scalograms and a deep convolutional neural network

Adam L. Halberstadt^1,2*^

*^1^ Department of Psychiatry, University of California San Diego, La Jolla, CA, USA*

*^2^ Research Service, VA San Diego Healthcare System, San Diego, CA, USA*

% This file includes step-by step instructions for training the Support Vector Machine (SVM)

% algorithm and saving it as an error-correcting output code (ECOC) classifier.

% This program is a MATLAB Live Code File. Execution in MATLAB requires the

% following toolboxes: Deep Learning Toolbox, Wavelet Toolbox,

% Signal Processing Toolbox, Statistics and Machine Learning Toolbox, Image

% Processing Toolbox, and Deep Learning Toolbox Model for ResNet-50 Network.

% The first step is to generate a set of scalograms that can be used to train the SVM.

% The main analysis program can be used to generate the scalograms. However, because the

% analysis program requires the ECOC model to run, the section of the program that performs

% the classification must be deleted until the ECOC classifier is trained.

% In addition to head twitches, examples of other types of activity should be present in the data

% set (e.g., jumping, seizures, ambulation, rearing, grooming, etc). The threshold for the

% *findpeaks()* function should be adjusted by changing the integer value in the line used to set

% the minht variable. In general, the threshold should be set so that the preprocessing step

% detects virtually all of the head twitches in the magnetometer recordings but does not detect a

% large number of non-HTR events. Although the error rate of classification is low, errors do

% occur, meaning that errors will accumulate if the preprocessing step identifies very large

% numbers of non-HTR events. Overall, most of the events identified in preprocessing should

% be head twitches. Once the dataset has been created, examine the detected events and divide

% them into “HTR” and “OTHER” categories. Then save all of the head twitches into the folder

% “\Training\HTR\”, and all the other events into the folder “\Training\OTHER\”. Make sure

% that both folders contain the same number of scalograms. Execute the following commands in

% MATLAB using “\Training\” as the current directory and then repeat the process, as

% necessary, until an acceptable level of performance is achieved.

% Once the SVM is trained, it can be saved as an ECOC by executing the following command:

% >> save HTR classifier

directory = pwd;

images = imageDatastore(directory, 'LabelSource', 'foldernames', 'IncludeSubfolders', true);

net = resnet50();

[trainingSet, testSet] = splitEachLabel(images, 0.25, 'randomize');

imageSize = net.Layers(1).InputSize;

augmentedTrainingSet = augmentedImageDatastore(imageSize, trainingSet, ...

'ColorPreprocessing', 'gray2rgb');

augmentedTestSet = augmentedImageDatastore(imageSize, testSet, ...

'ColorPreprocessing', 'gray2rgb');

featureLayer = 'fc1000';

trainingFeatures = activations(net, augmentedTrainingSet, featureLayer, ...

'MiniBatchSize', 10, 'OutputAs', 'columns');

trainingLabels = trainingSet.Labels;

classifier = fitcecoc(trainingFeatures, trainingLabels, ...

'Learners', 'Linear', 'Coding', 'onevsall', 'ObservationsIn', 'columns');

testFeatures = activations(net, augmentedTestSet, featureLayer, ...

'MiniBatchSize', 10, 'OutputAs', 'columns');

predictedLabels = predict(classifier, testFeatures, 'ObservationsIn', 'columns');

testLabels = testSet.Labels;

confMat = confusionmat(testLabels, predictedLabels);

confMat = bsxfun(@rdivide,confMat,sum(confMat,2))
